# Supplementary material for: The cytoplasmic nuclear receptor RARγ controls RIP1 initiated cell death when cIAP activity is inhibited
Source: Nat Commun. 2017 Sep 4;8:425. doi: 10.1038/s41467-017-00496-6 (PMC5583178; doi:10.1038/s41467-017-00496-6)
Supplement: Supplementary file 1 — Supplementary Information [file 41467_2017_496_MOESM1_ESM.pdf]

File Name: Peer Review File

File Name: Supplementary Information
Description: Supplementary Figures.

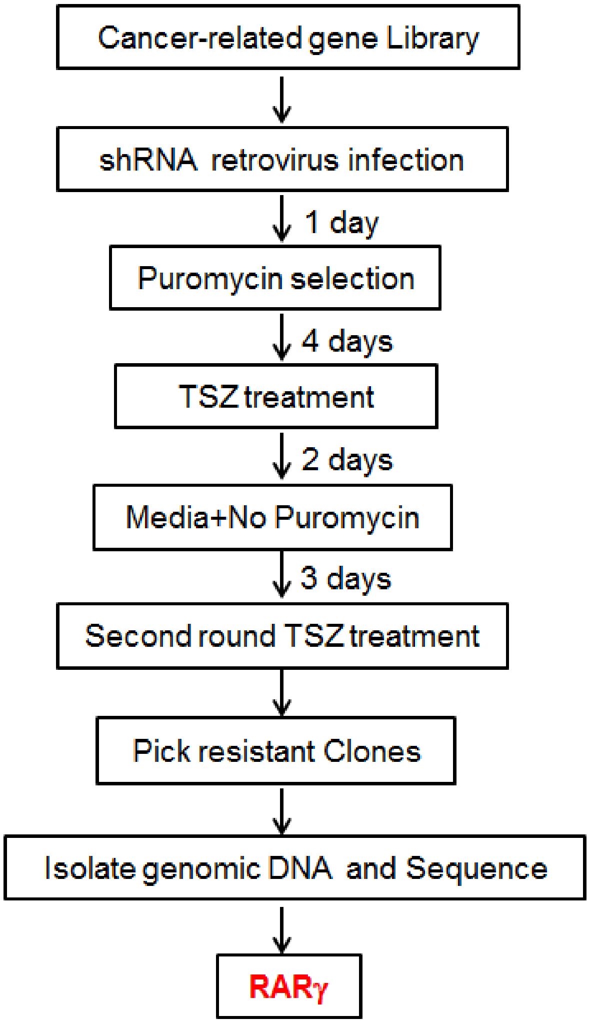

Supplementary Figure 1. Scheme for the Identification of RARγ

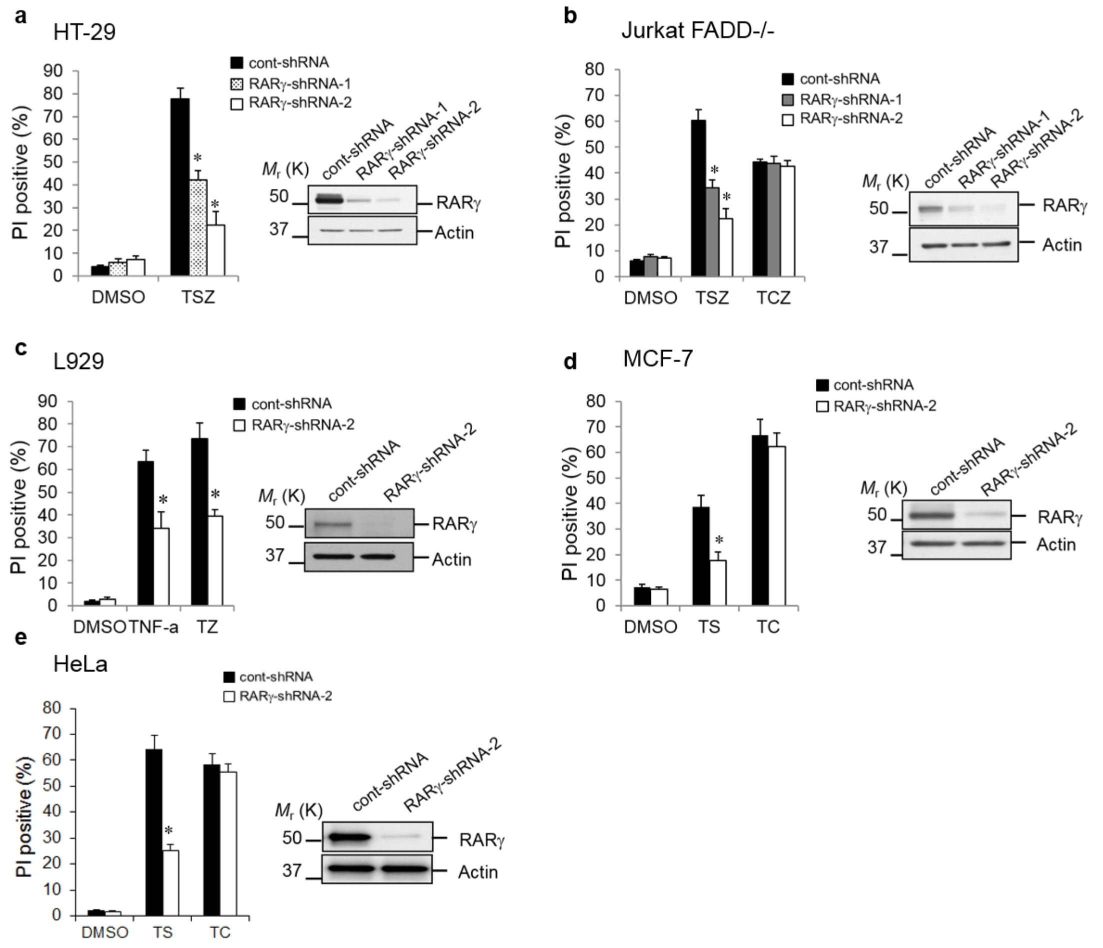

Supplementary Figure 2. RARγ is required for apoptosis and necroptosis in different cell types. (a-b) HT-29 or Jurkat FADD-/- cells stably expressing cont-shRNA, RARγ-shRNA-1, or RARγ-shRNA-2 were treated with DMSO or TSZ or TCZ for 24 hr. Cell death was determined by PI staining using flow cytometry (left panel). The expression levels of RARγ and Actin were detected by immunoblotting (right panel). (c) L929 cells expressing cont-shRNA or RARγ-shRNA-2 were treated with DMSO, TNF-α for 24h or TZ for 4 hr. Cell death was determined by PI staining using flow cytometry (left panel). The expression levels of RARγ and Actin were detected by immunoblotting (right panel). (d-e) MCF-7 or HeLa cells expressing cont-shRNA or RARγ-shRNA-2 were treated with DMSO or TS or TC for 24 hr. Cell death was determined by PI staining using flow cytometry (left panel). The expression levels of RARγ and Actin were detected by immunoblotting (right panel). (The statistical analysis for a-e, * $p < 0.05$ versus cont-shRNA; ANOVA for a,b; Student's t-text for c-e). The bars represent the mean±s.e.m. of three experiments. All blots above are representative of one of three experiments.

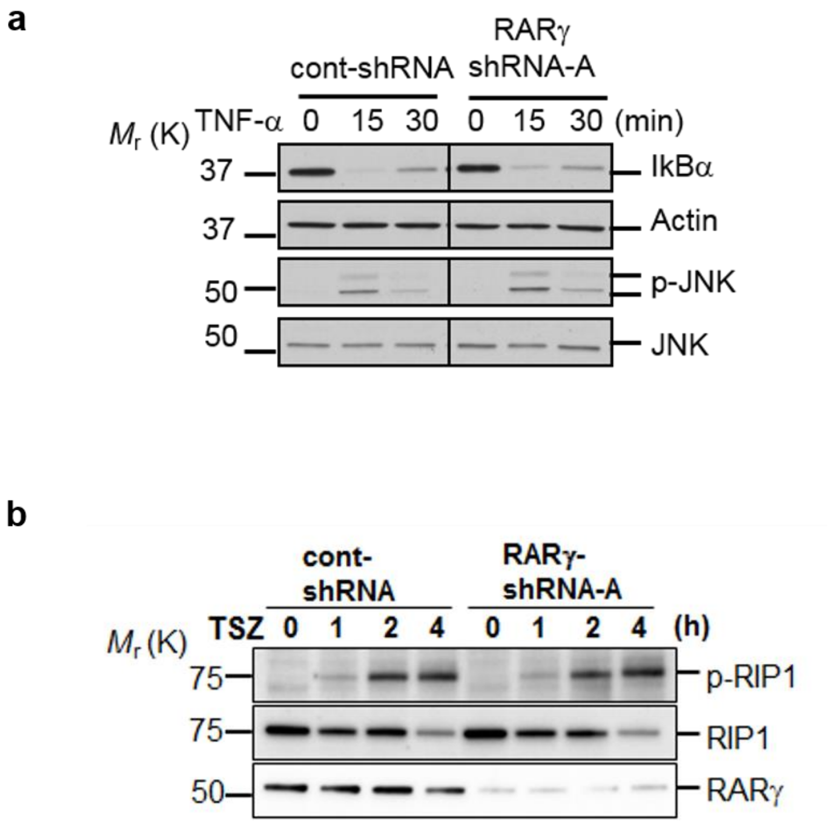

Supplementary Figure 3. Loss of RARγ does not affect TNFα-induced activation of NF-κB and JNK and TSZ-induced RIP1 autophosphorylation. (a-b) Western blot analysis of HT-29 cont-shRNA and RARγ-shRNA-A cells treated with TNF-α (a) or TSZ (b) for the indicated times. Cell lysates were immunoblotted with the indicated antibodies. All blots above are representative of one of three experiments.

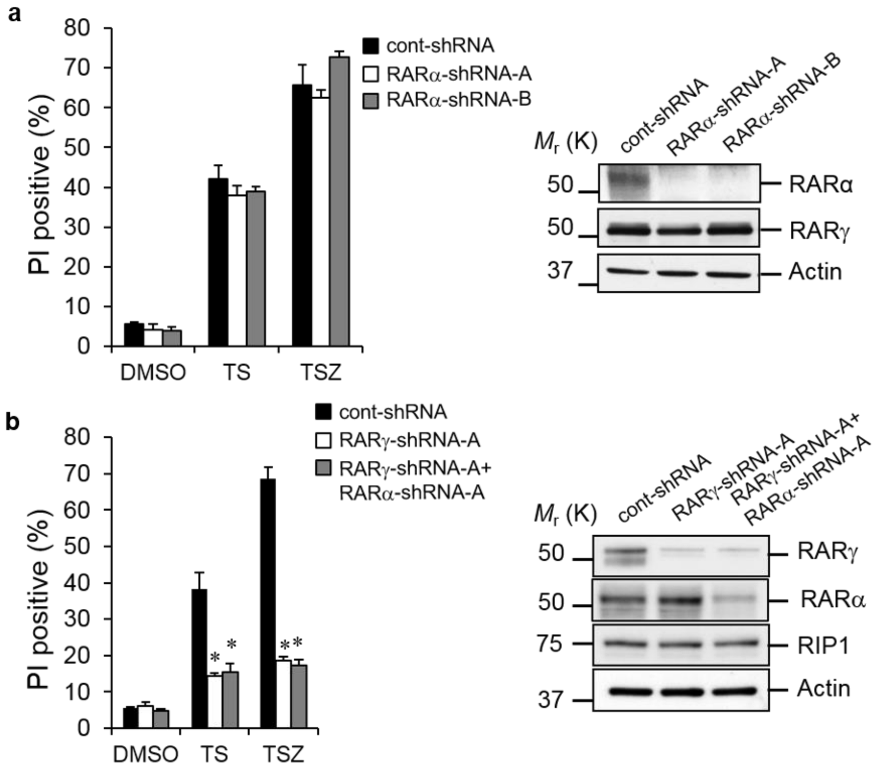

Supplementary Figure 4. RARα is not required for necroptosis. (a) Cell death analysis of HT-29 cells infected with cont-shRNA, RARα-shRNA-A, or RARα-shRNA-B and treated with TS or TSZ for 24 hr. PI positive population was determined by flow cytometry (left panel). The expression levels of RARα, RARγ and Actin were detected by immunoblotting (right panel). (b) Cell death analysis of HT-29 cells infected with cont-shRNA, RARγ-shRNA-A, or RARγ-shRNA-A and RARα-shRNA-A and treated with TS or TSZ for 24 hr. PI positive population was determined by flow cytometry (left panel). The expression levels of RARα, RARγ, RIP1 and Actin were detected by immunoblotting (right panel). (The statistical analysis for a and b, * $p < 0.05$ versus cont-shRNA; ANOVA). The bars represent the mean±s.e.m. of three experiments. All blots above are representative of one of three experiments.

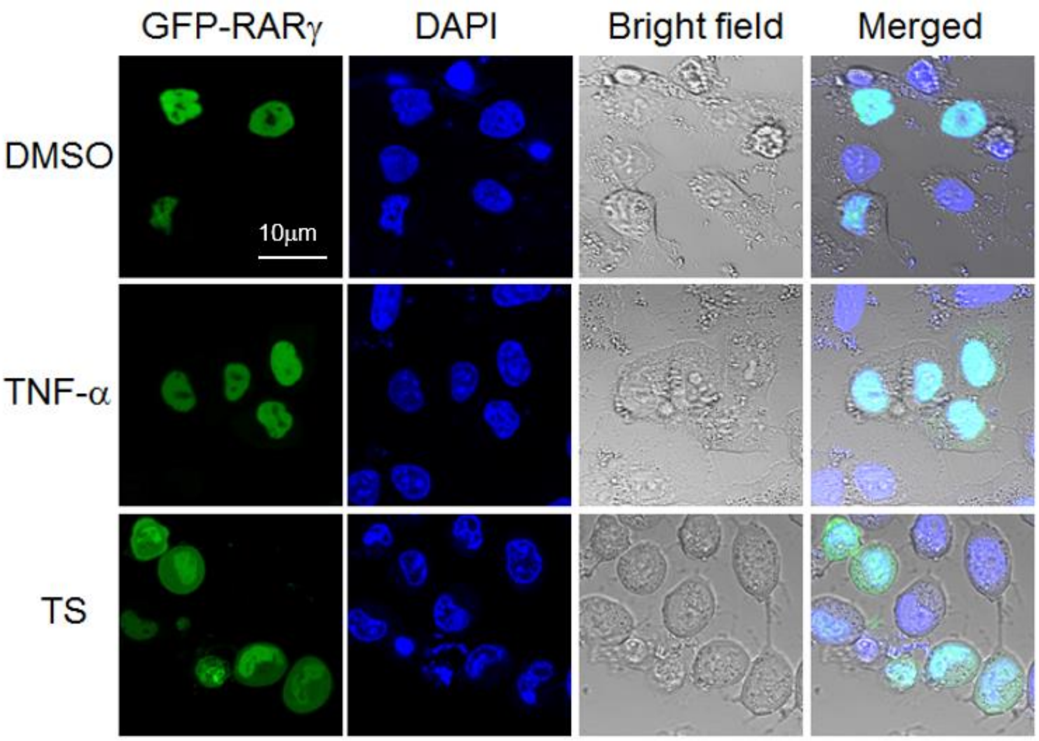

Supplementary Figure 5. TS treatment induced cytosolic RARγ localization. Confocal microscopy of HeLa cells transfected with RARγ-GFP plasmid and treated with DMSO, TNF-α, or TS for 2 hr. (blue: DAPI; green: RARγ). (bar: 10μm). Images are representative of one of three experiments.

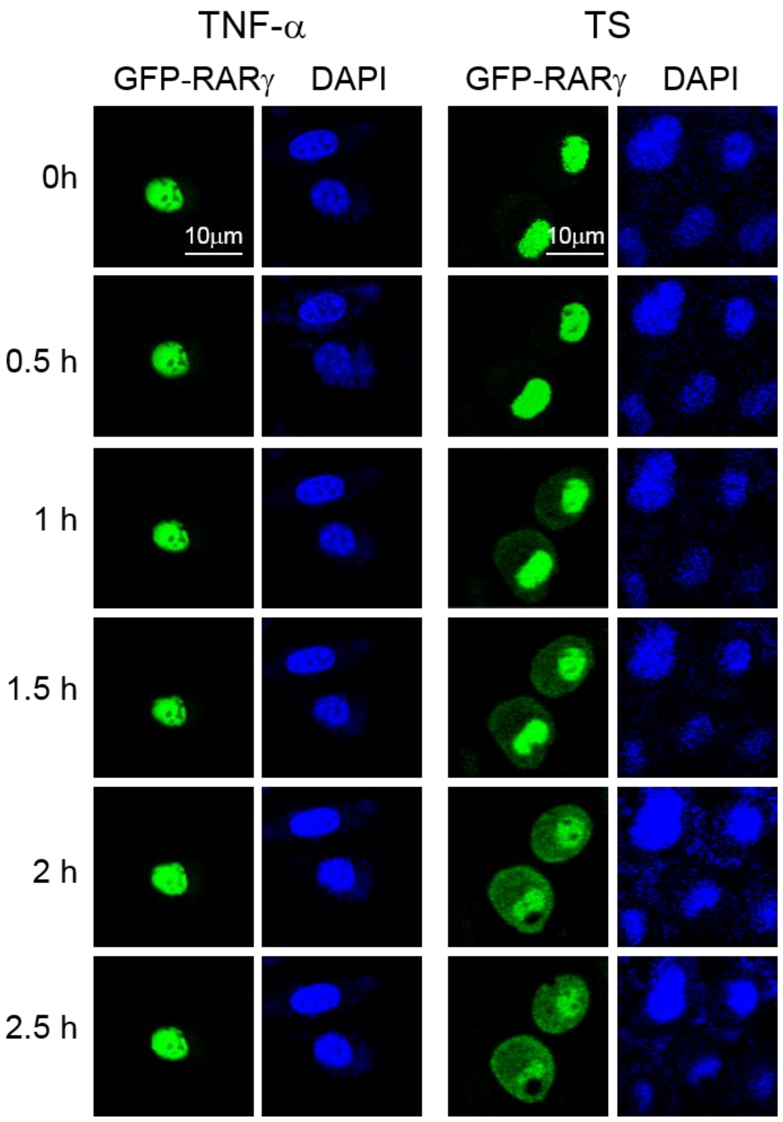

Supplementary Figure 6. Time-lapse imaging of RARγ nuclear localization. Time-lapse confocal microscopy of HeLa cells transfected with GFP-RARγ plasmid and treated with TNF-α or TNF-α and Smac-mimetic together (TS). Images were done at the indicated time points. (blue: DAPI; green: RARγ). (bar: 10μm). Images are representative of one of three experiments.

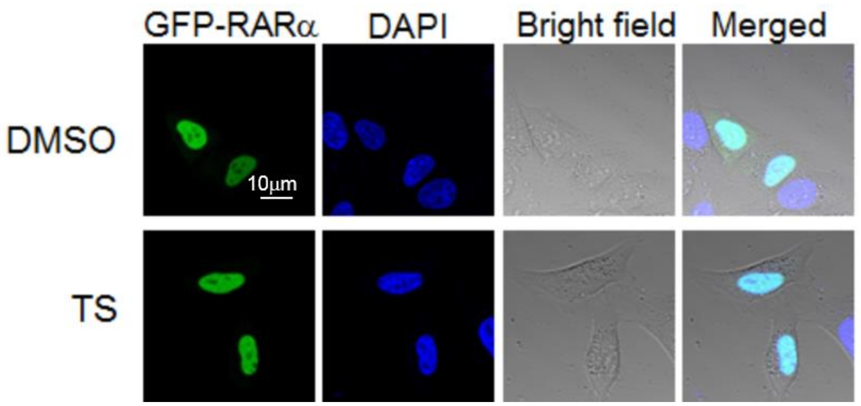

Supplementary Figure 7. Nuclear localization of RARα. Confocal microscopy of HeLa cells transfected with GFP-RARα plasmid and treated with DMSO or TS for 2hr. (blue: DAPI; green: RARα). (bar: 10μm). Images are representative of one of three experiments.

a

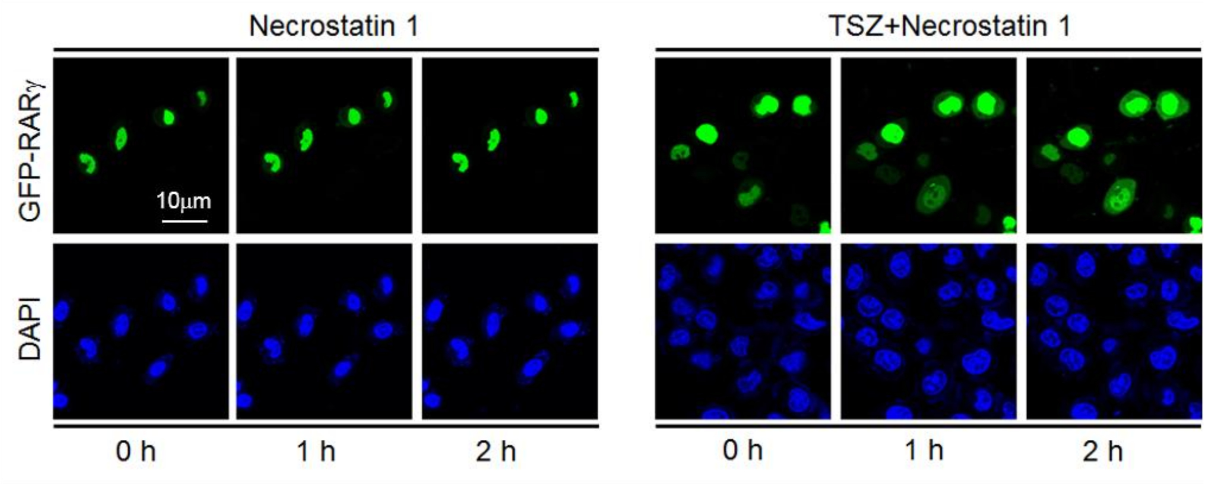

b

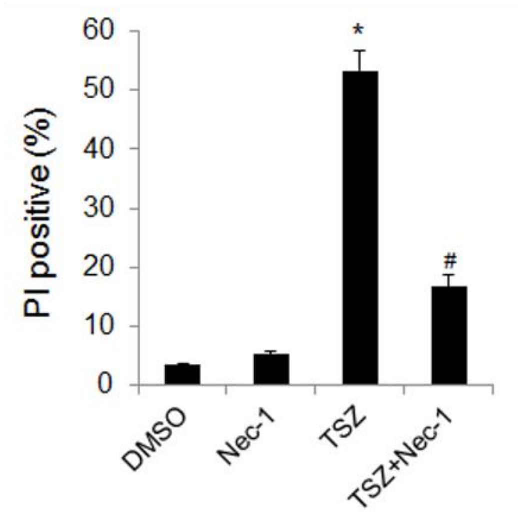

Supplementary Figure 8. Necrostain-1 block RARγ induced RIP1 medicated cell death. (a) Confocal microscopy of HeLa reconstituted with RIP3 (HeLa-RIP3) were transfected with GFP-RARγ plasmid and treated with Necrostatin-1 or TSZ and Necrostatin-1 together. Pictures were shown as indicated time points. (blue: DAPI; green: RARγ). (bar: 10μm). Images are representative of one of three experiments. (b) Cell death analysis of HeLa-RIP3 cells treated with DMSO or Necrostain-1 for 1hr and then treated with or without TSZ for 24 hr. PI positive population was determined by flow cytometry. (* $p < 0.05$ versus DMSO. # $p < 0.05$ versus TSZ; ANOVA). The bars represent the mean±s.e.m. of three experiments.

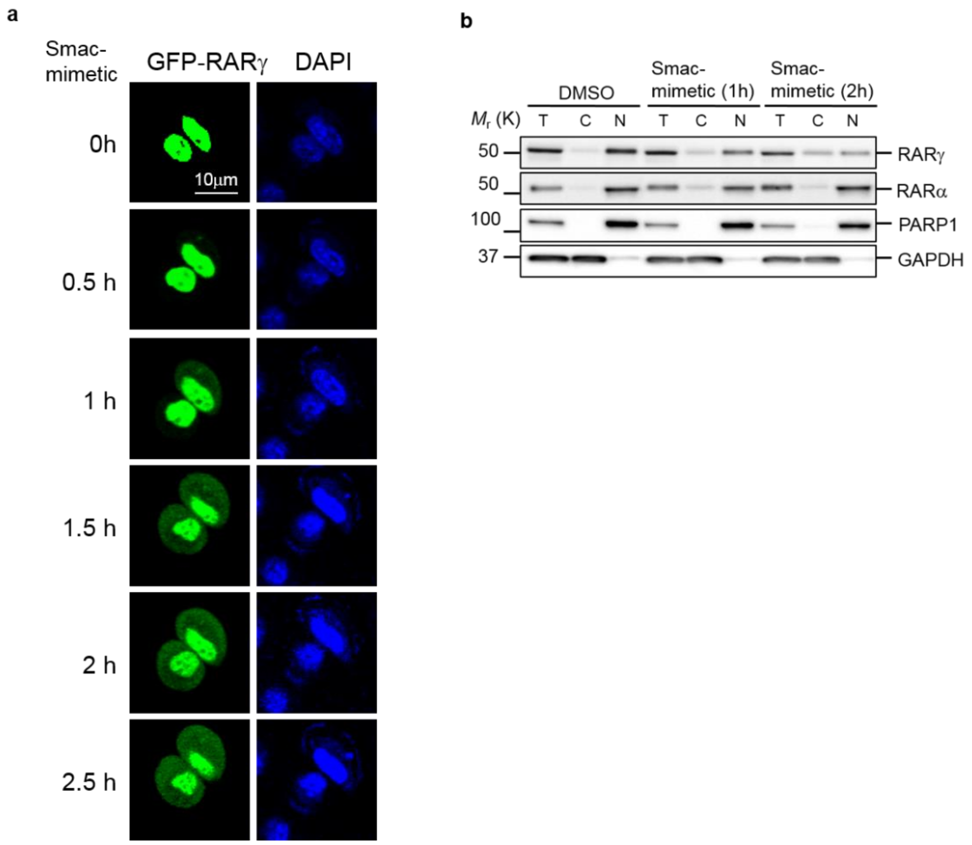

Supplementary Figure 9. Time-lapse imaging of RARγ nuclear localization. (a) Time-lapse confocal microscopy of HeLa cells transfected with GFP-RARγ plasmid and treated with Smac-mimetic. Images were done at the indicated time points. (blue: DAPI; green: RARγ). (b) The total (T), cytosolic (C), and nuclear (N) fractions analysis of HeLa cells treated with DMSO, or Smac-mimetic for 1 or 2 hr. Fractions were analyzed by immunoblotting with the indicated antibodies. (bar: 10μm). All images and blots above are representative of one of three experiments.

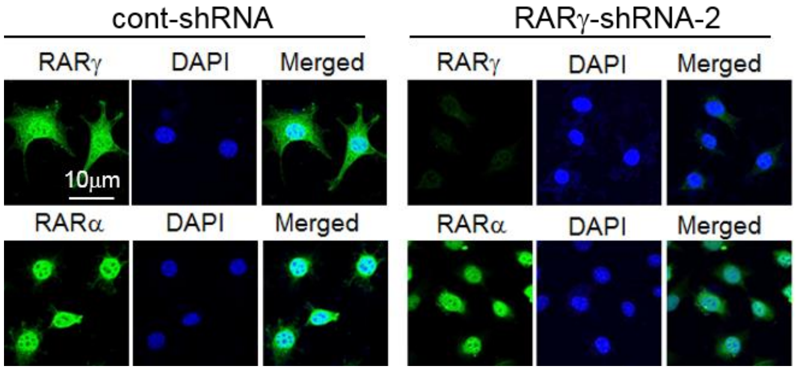

Supplementary Figure 10. Localization of endogenous RARγ and RARα in L929 cells. The localization of RARγ and RARα in L929 cells expressing cont-shRNA or RARγ-shRNA-2 were assessed by Immunofluorescent staining. (bar: 10μm). Images are representative of one of three experiments.

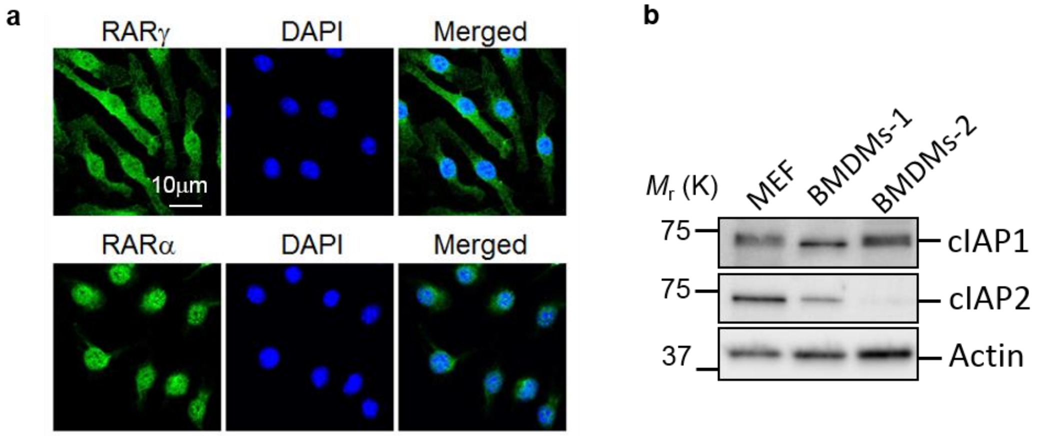

Supplementary Figure 11. Localization of endogenous RARγ and RARα and cIAP1/2 expression in BMDMs. (a)The localization of endogenous RARγ and RARα in BMDMs were assessed by Immunofluorescent staining. (bar: 10μm). (b)The expression of cIAP1 and cIAP2 in BMDMs were tested by immunoblotting using indicated antibody. All images and blots above are representative of one of three experiments.

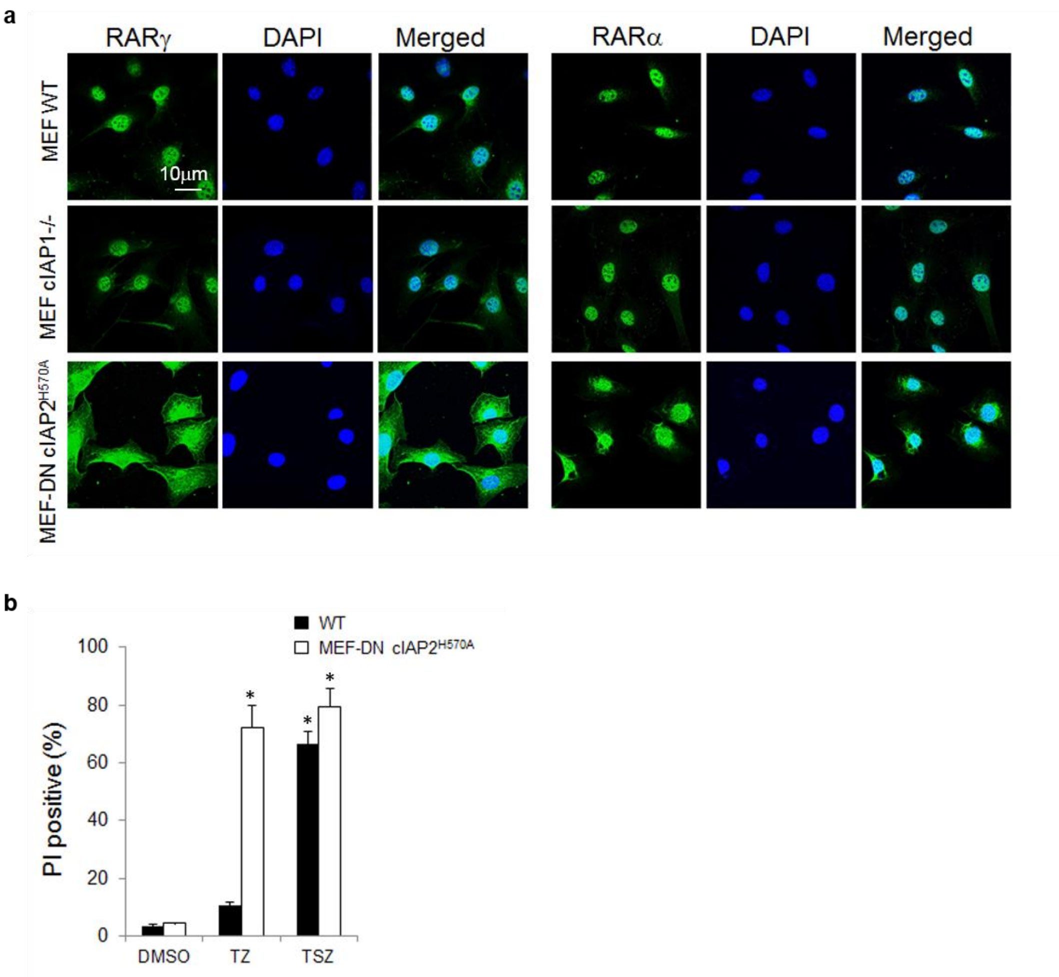

Supplementary Figure 12. Localization of endogenous RARγ and RARα in MEFs. (a) The localization of RARγ endogenous and RARα in MEF WT, cIAP1-/- and dominant negative (DN)-cIAP2^{H570A} were assessed by Immunofluorescent staining. (bar: 10μm). Images are representative of one of three experiments. (b) MEF WT and dominant negative (DN)-cIAP2^{H570A} treated with DMSO, TZ or TSZ for 24h. Cell death was determined by PI staining using flow cytometry. (* $p < 0.05$ versus DMSO; ANOVA). The bars represent the mean±s.e.m. of three experiments.

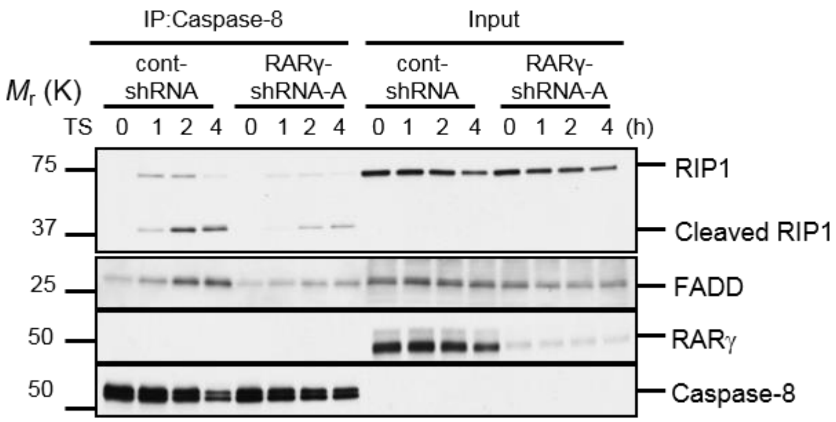

Supplementary Figure 13. RARγ affects the formation of complex II. HT-29 cont-shRNA and RARγ-shRNA-A treated with TS for the indicated times. Cell lysates were immunoprecipitated with anti-Caspase 8 antibody and analyzed with the indicated antibodies. Blots are representative of one of three experiments.

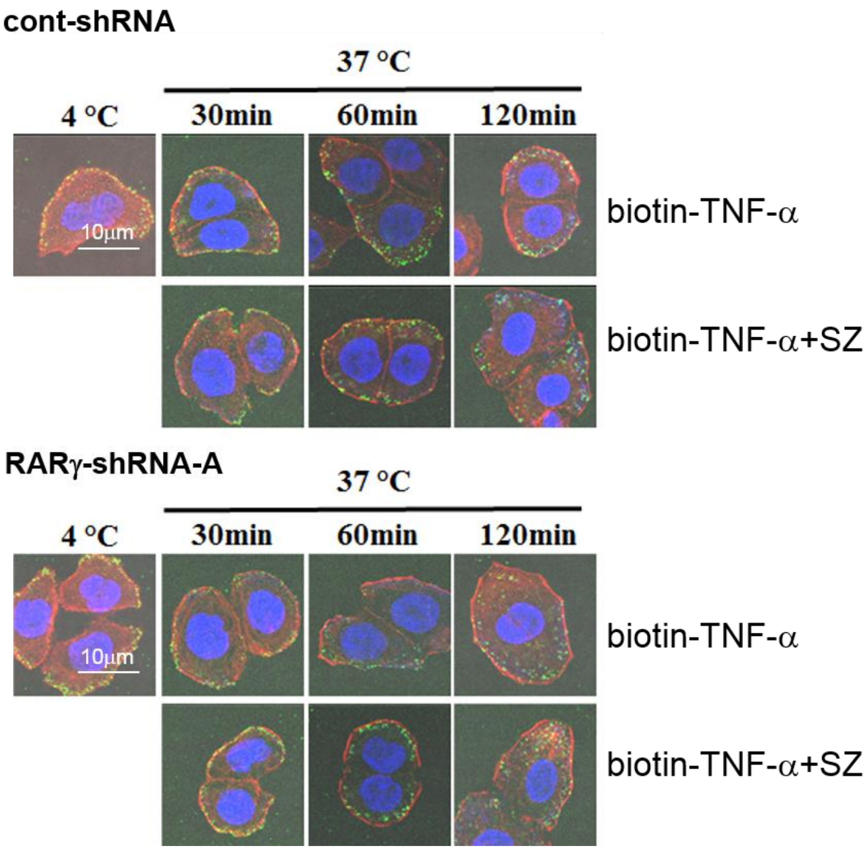

Supplementary Figure 14. RARγ does not affects TNFR1 internalization. HT-29 cont-shRNA and RARγ-shRNA-A treated with biotin-TNF coupled to streptavidin-FITC in the presence of SZ. Confocal Images were done at the indicated time points. (blue: DAPI; green: TNF receptors; red: F-actin). (bar: 10μm). Images are representative of one of three experiments.

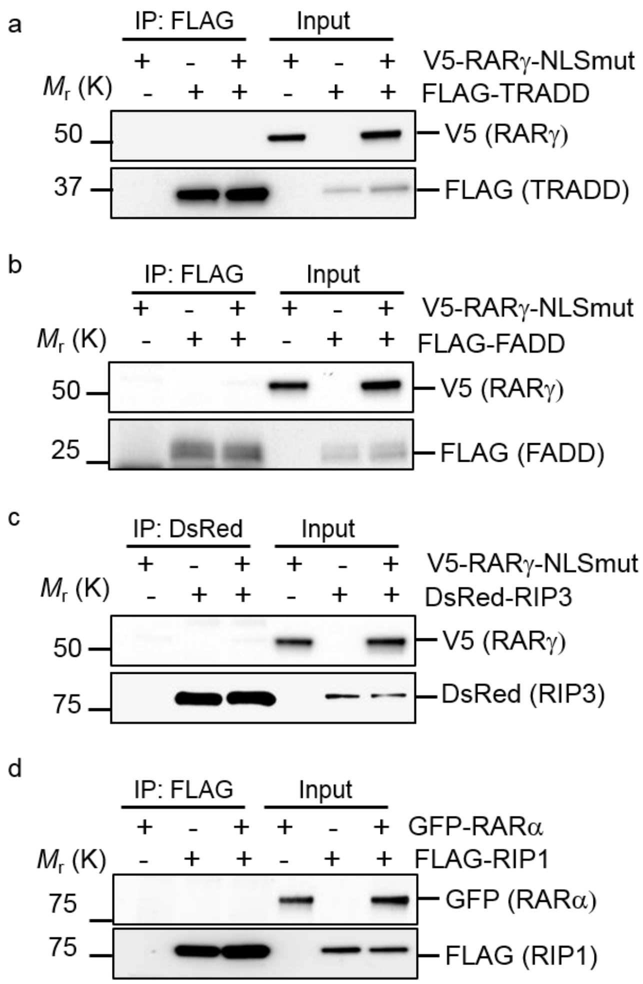

Supplementary Figure 15. RARγ does not interact with TRADD, FADD, or RIP3 and RARα does not interact with RIP1. (a-c) HEK293T cells were co-transfected with V5-RARγ-NLSmut and with or without FLAG-TRADD (a), or FLAG-FADD (b), or DsRed-RIP3 (c) plasmids as indicated. Cell lysates were immunoprecipitated using anti-FLAG (TRADD) (a), anti-FLAG (FADD) (b), or anti-DsRed (c) antibody. (d) HEK293T cells were co-transfected with GFP-RARα and FLAG-RIP1 plasmids. Cell lysates were immunoprecipitated using anti-FLAG (RIP1). The immunoprecipitated complex was analyzed by immunoblotting as indicated. The blots above are representative of one of three experiments.

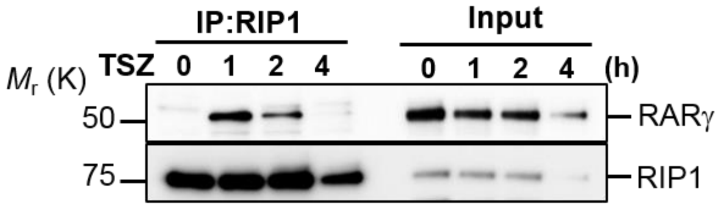

Supplementary Figure 16. RIP1 interacted with RARγ under TSZ treatment. HT-29 cells were treated with TSZ for the indicated times. Cell lysates were immunoprecipitated with anti-RIP1 antibody and analyzed by immunoblotting with the indicated antibodies. Blots are representative of one of three experiments.

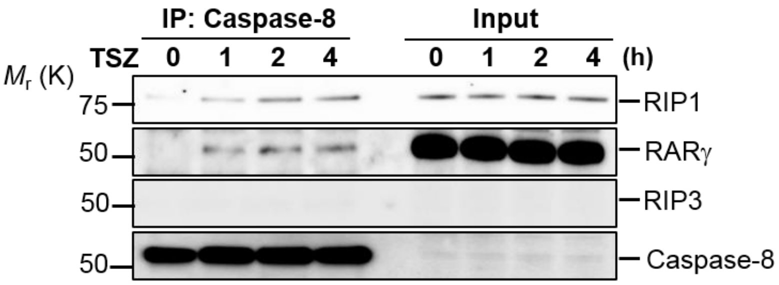

Supplementary Figure 17. RIP1 and RARγ are in complex IIa. HeLa cells were treated with TSZ for the indicated times. Cell lysates were immunoprecipitated with anti-Caspase-8 antibody and analyzed by immunoblotting with the indicated antibodies. Blots are representative of one of three experiments.

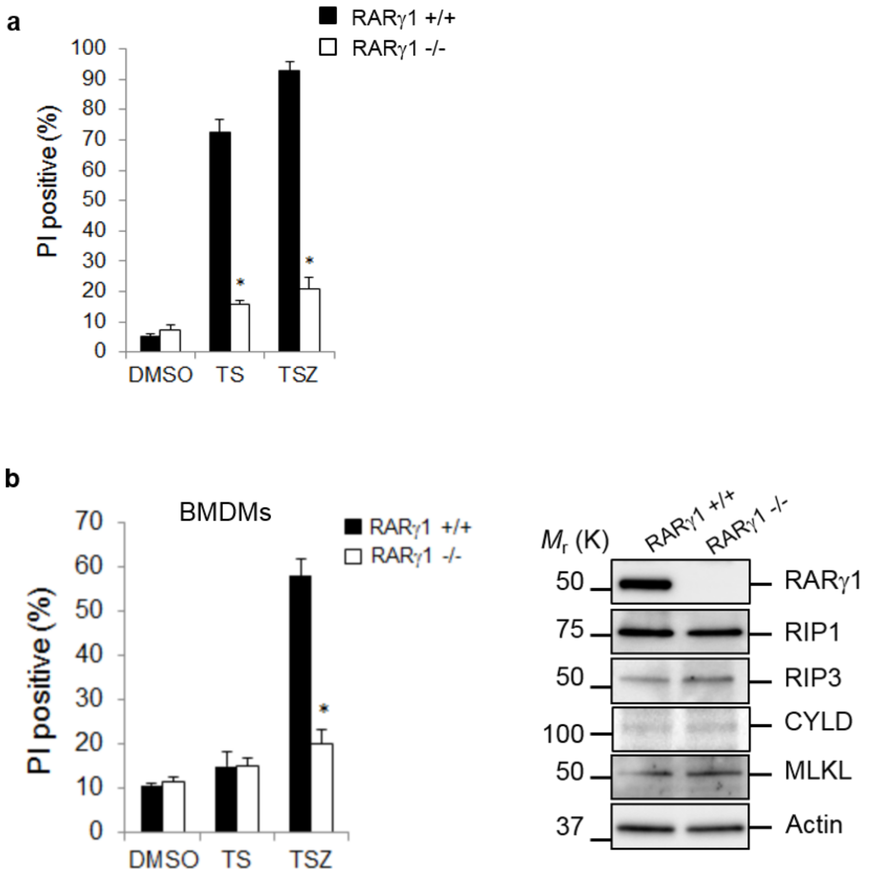

Supplementary Figure 18. RARγ protected MEFs and BMDMs against TNF-Induced death. (a) Immortalized RARγ1 +/+ and RARγ1 -/- MEFs were treated with DMSO, TS or TSZ for 24 h. Cell death was determined by PI staining using flow cytometry. (* $p < 0.05$ versus RARγ1 +/+; ANOVA). The bars represent the mean±s.e.m. of three experiments. (b) RARγ1 +/+ and RARγ1 -/- BMDMs were treated with DMSO, TS or TSZ for 24 h. Cell death was determined by PI staining using flow cytometry (left panel) (* $p < 0.05$ versus RARγ1 +/+; ANOVA). The bars represent the mean±s.e.m. of three experiments. Cell was analyzed by immunoblotting using indicated antibody (right panel). Blots are representative of one of three experiments.

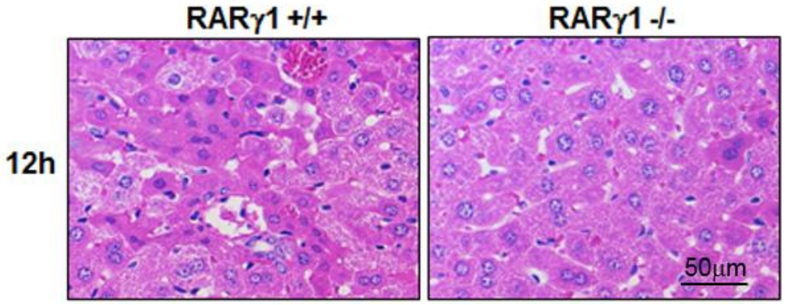

Supplementary Figure 19. RARγ1-/- mice are protected against TNF-induced death. Representative histologic livers were excised from RARγ1 +/+ and RARγ1 -/- mice 12 hours after TZ treatment. H&E staining of liver shows focal necrotic cells and red blood cells (bar: 50μm).

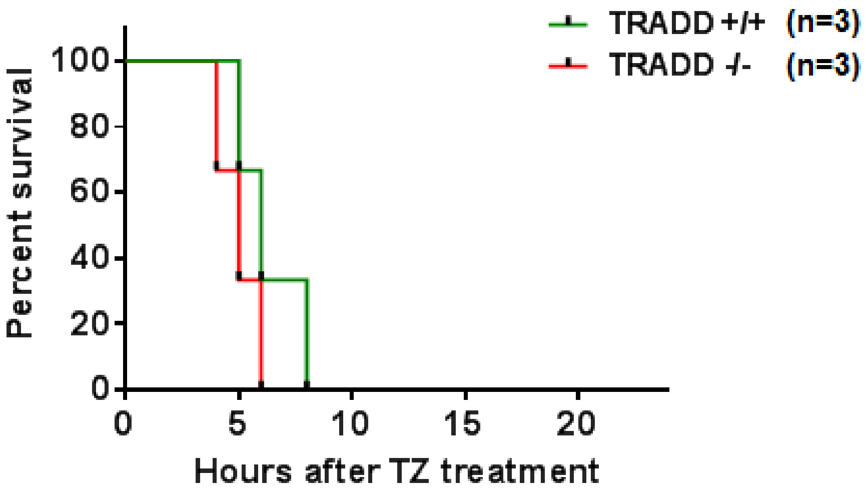

Supplementary Figure 20. TRADD does not protect mice against TZ-Induced death.
Survival curve of wild-type (green: TRADD +/+) and TRADD-knock-out (red: TRADD -/-) mice after treatment with z-VAD-fmk and TNF-α (TZ). (no significance; log rank test).

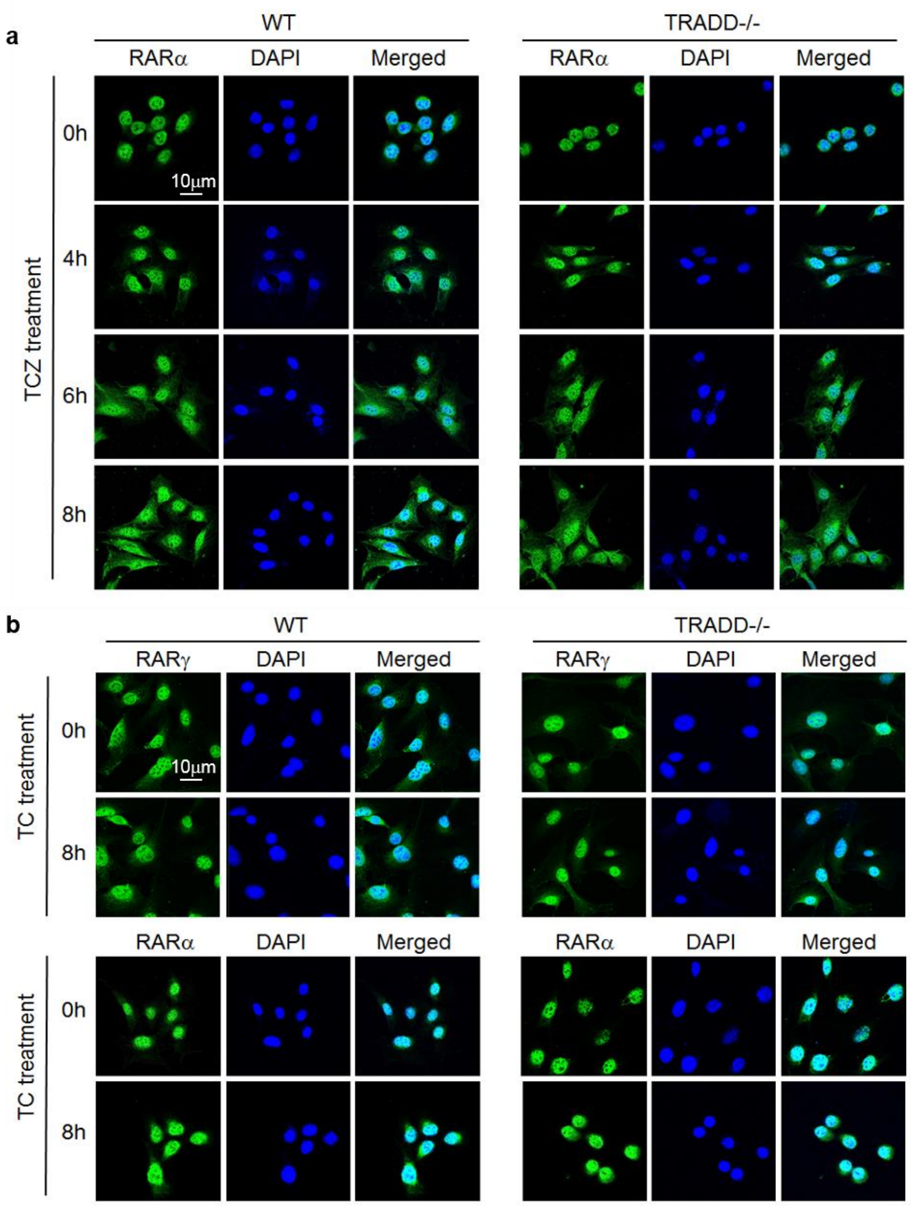

Supplementary Figure 21. TCZ, not TC, treatment induced cytosolic RARα and RARγ localization. (a-b) WT and TRADD -/- MEFs treated with TCZ (a) or TC (b) in indicated time points. The localization of endogenous RARα and RARγ assessed by Immunofluorescent staining (blue: DAPI; green: RARγ). (bar: 10μm). Images are representative of one of three experiments.

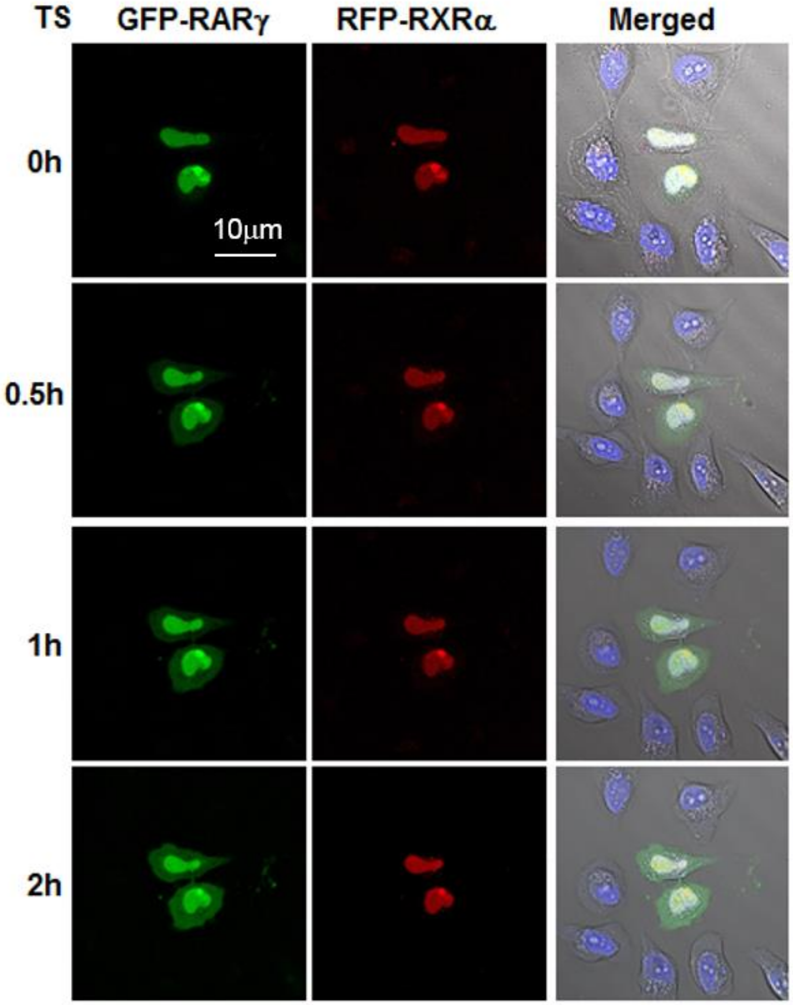

Supplementary Figure 22. RXRα did not affect RARγ localization in HeLa. Time-lapse confocal microscopy of HeLa cells that were co-transfected with GFP-RARγ and RFP-RXRα plasmids and treated with TS. Images were done at the indicated time points. (blue: DAPI; green: RARγ; red: RXRα). (bar: 10μm). Images are representative of one of three experiments.

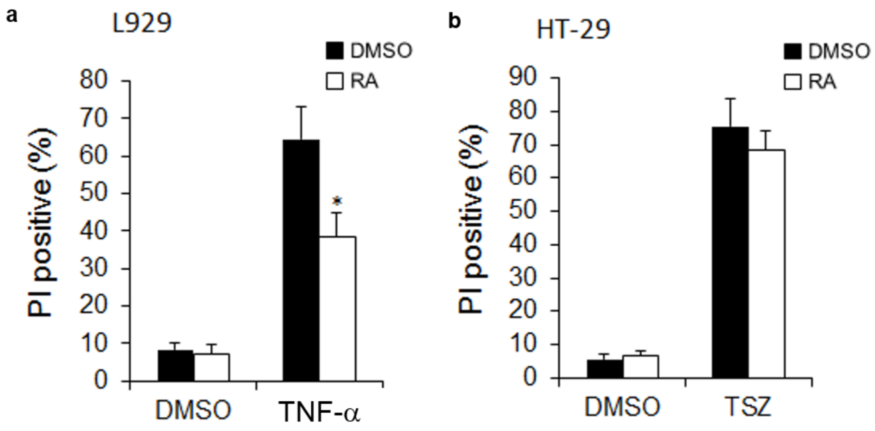

Supplementary Figure 23. Retinoic Acid protects L929, but not HT-29. L929 (left panel) or HT-29 (right panel) cells were treated with DMSO, or Retinoic Acid (RA) for 1hr and then treated with DMSO, TNF-α or TSZ. Cell death was determined by PI staining using flow cytometry. (* $p < 0.05$ versus DMSO; ANOVA). The bars represent the mean±s.e.m. of three experiments

Figure 1a

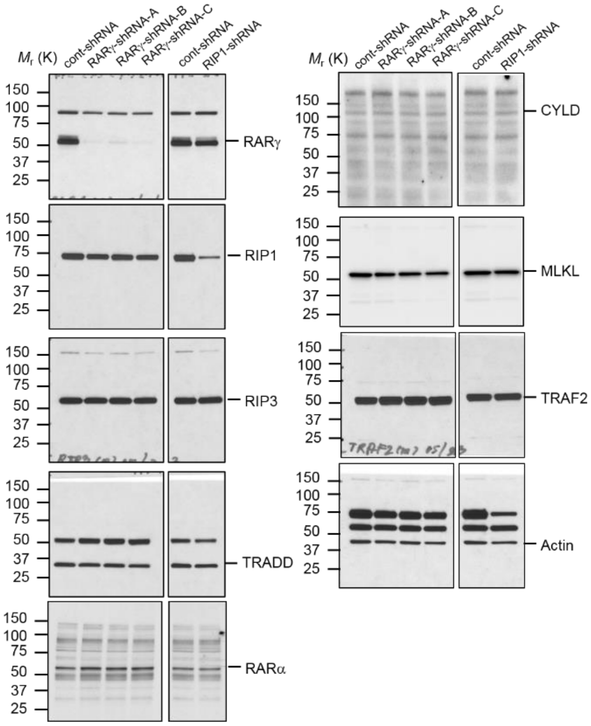

Figure 1b

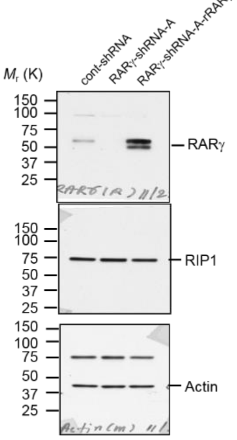

Figure 1e

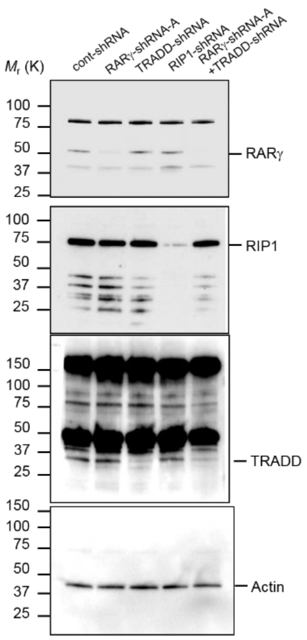

Figure 2b

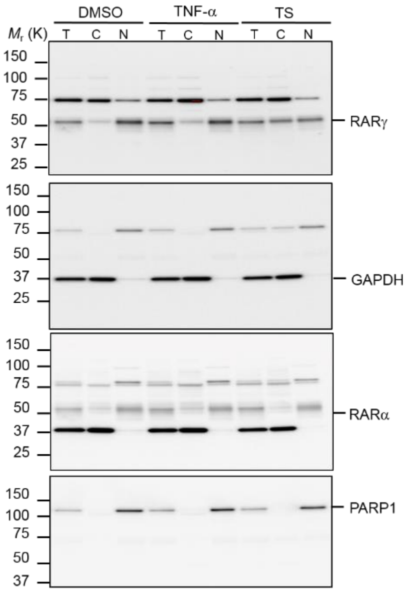

Figure 2e

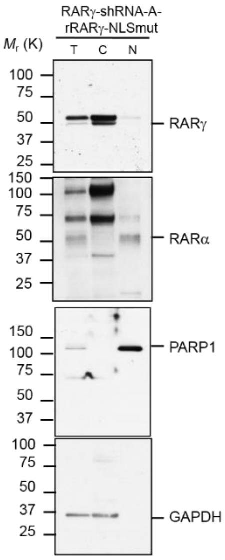

Figure 3a

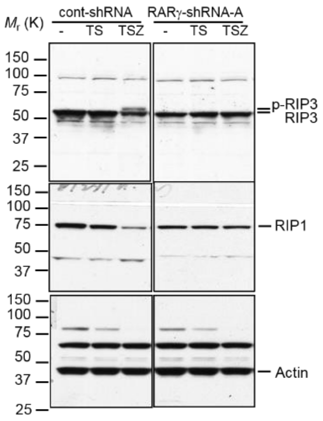

Supplementary Figure 24. Original immunoblotting of main figures

Figure 3b

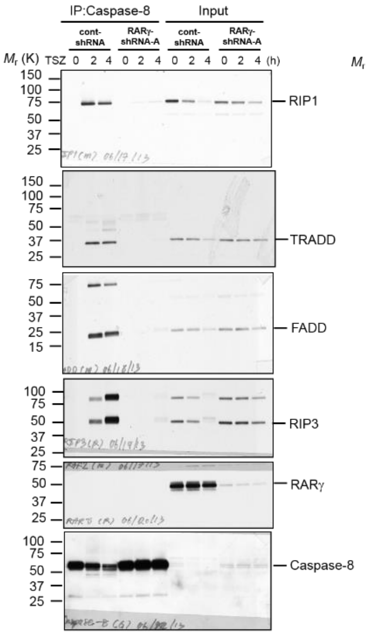

Figure 3c

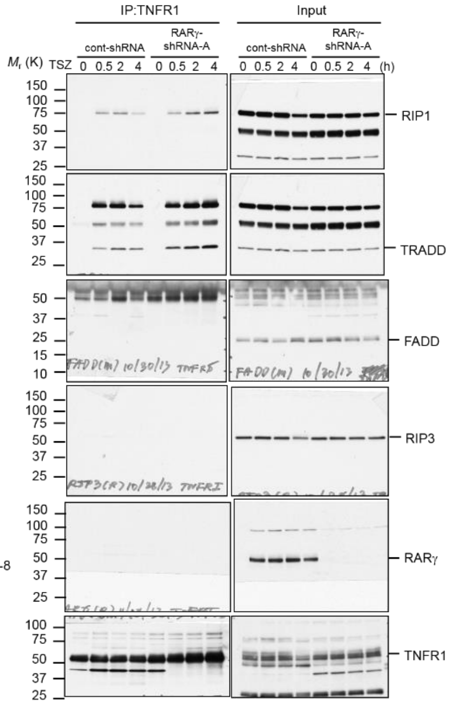

Figure 3d

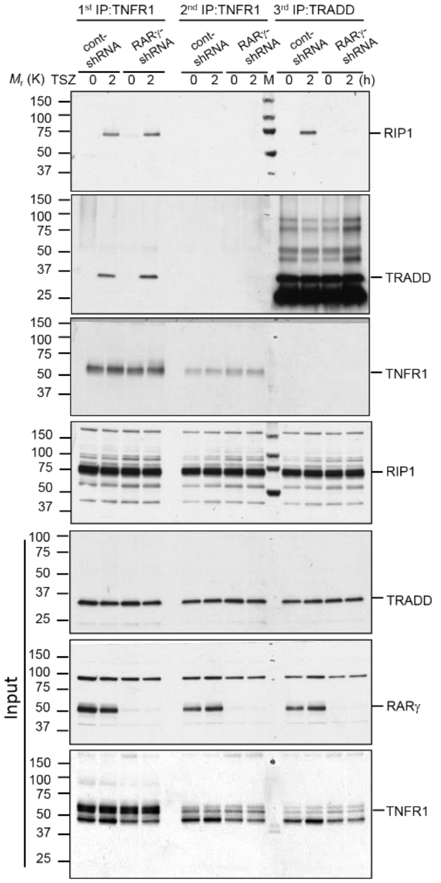

Figure 4a

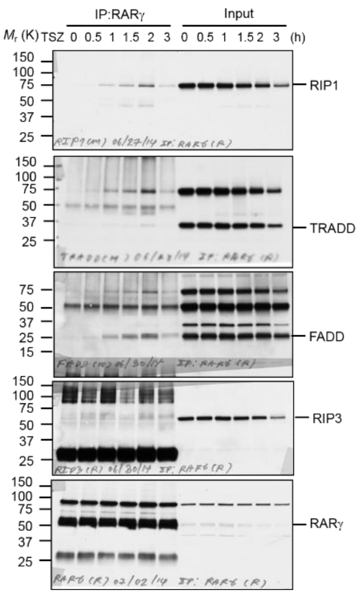

Figure 4c

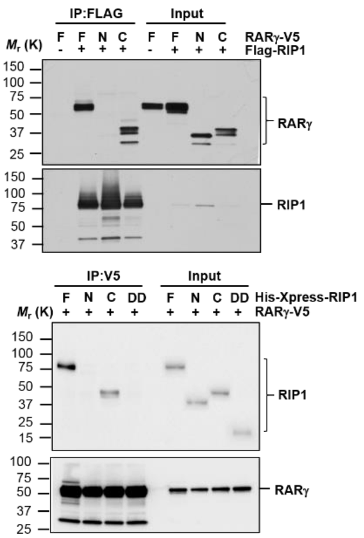

Figure 4b

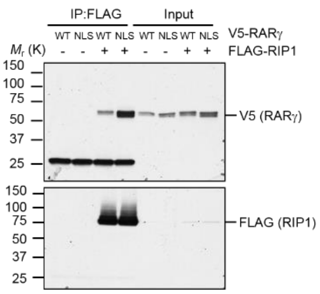

Supplementary Figure 25. Original immunoblotting of main figures

Figure 4d

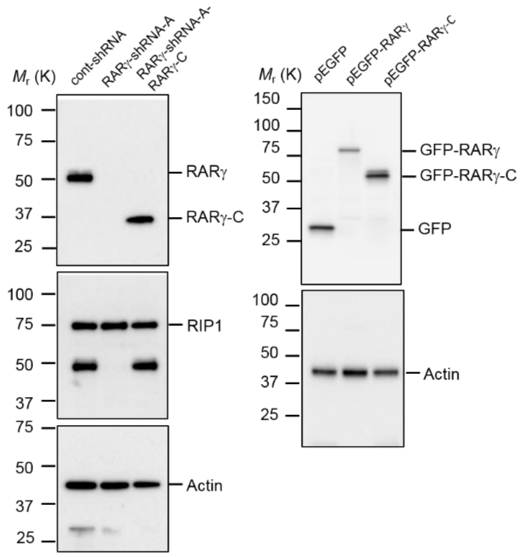

Figure 5a

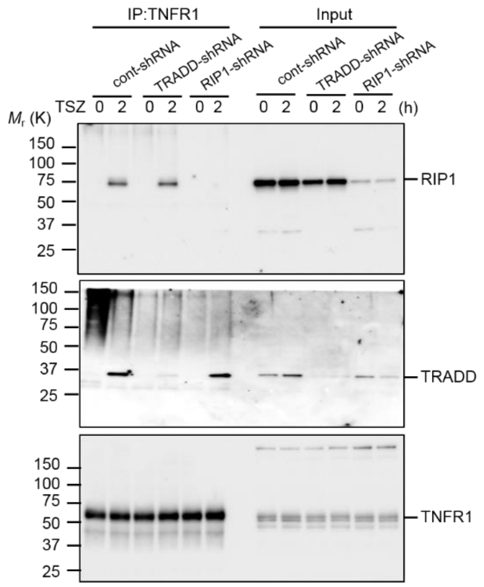

Figure 5b

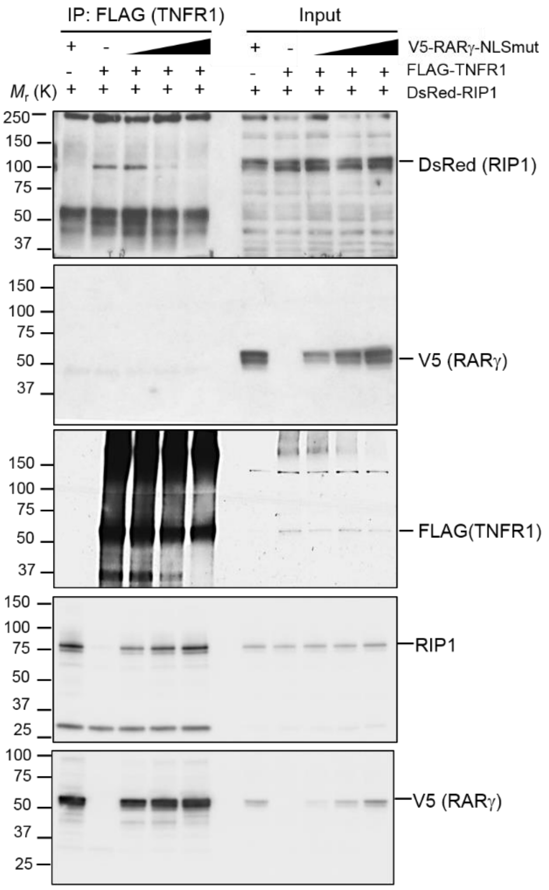

Figure 5c

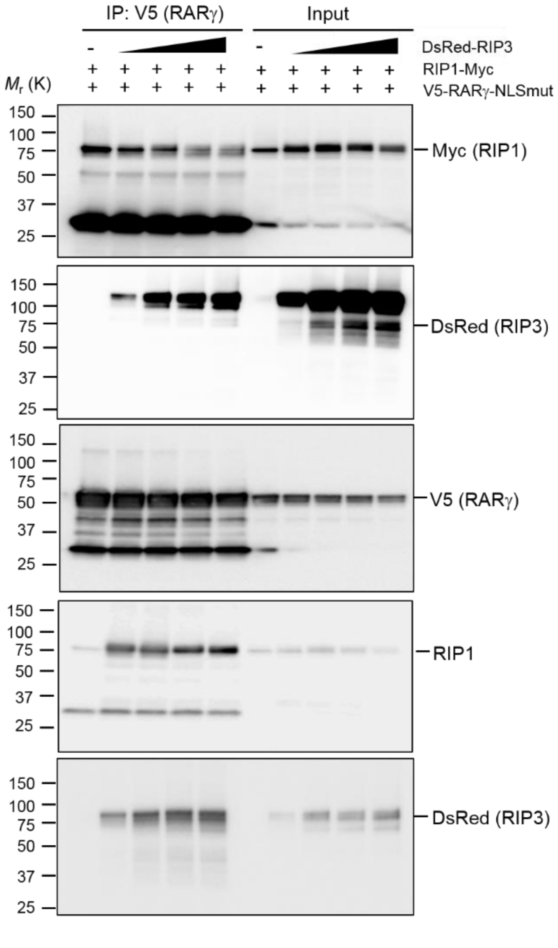

Supplementary Figure 26. Original immunoblotting of main figures

Figure 5d

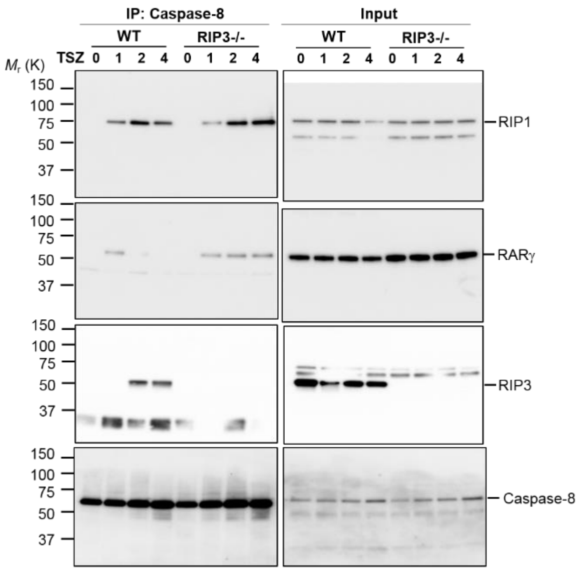

Figure 6b

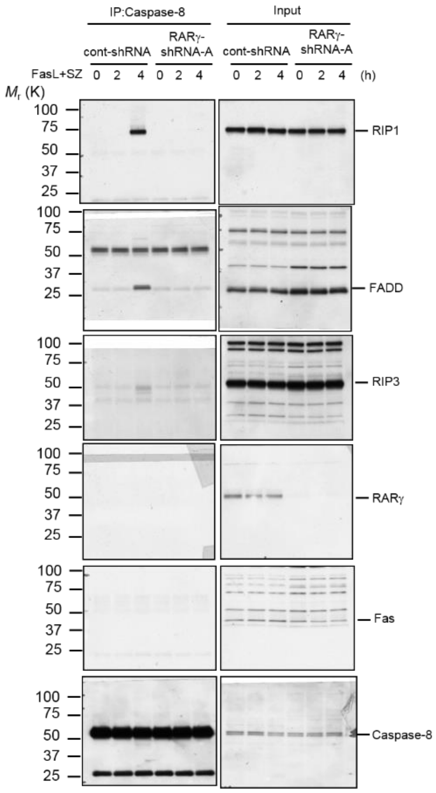

Figure 7c

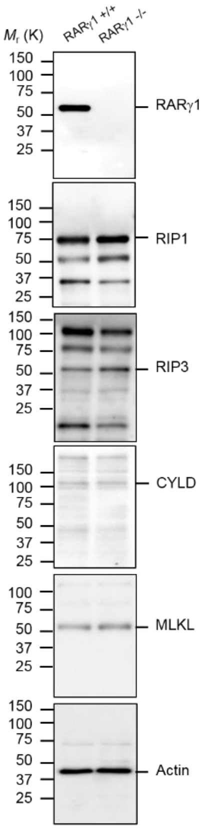

Figure 8a

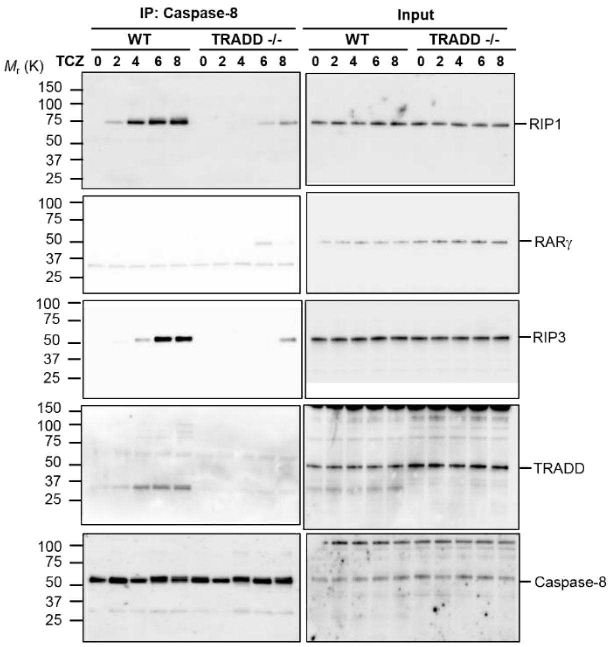

Figure 8b

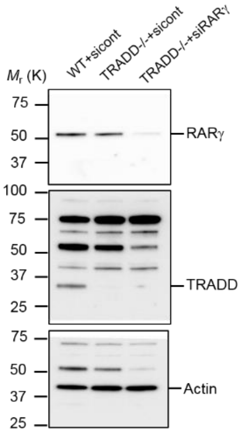

Supplementary Figure 27. Original immunoblotting of main figures

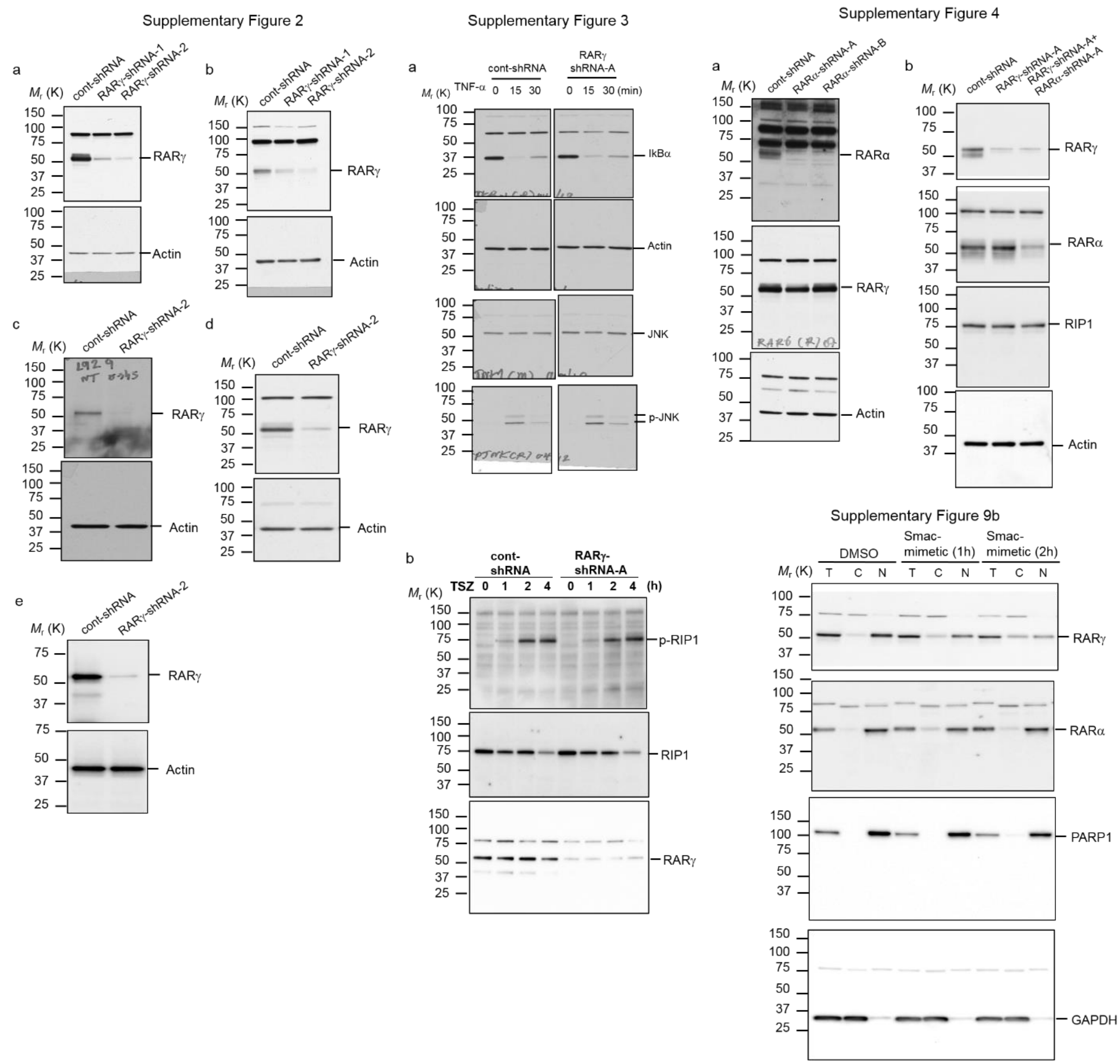

Supplementary Figure 28. Original immunoblotting of supplementary figures

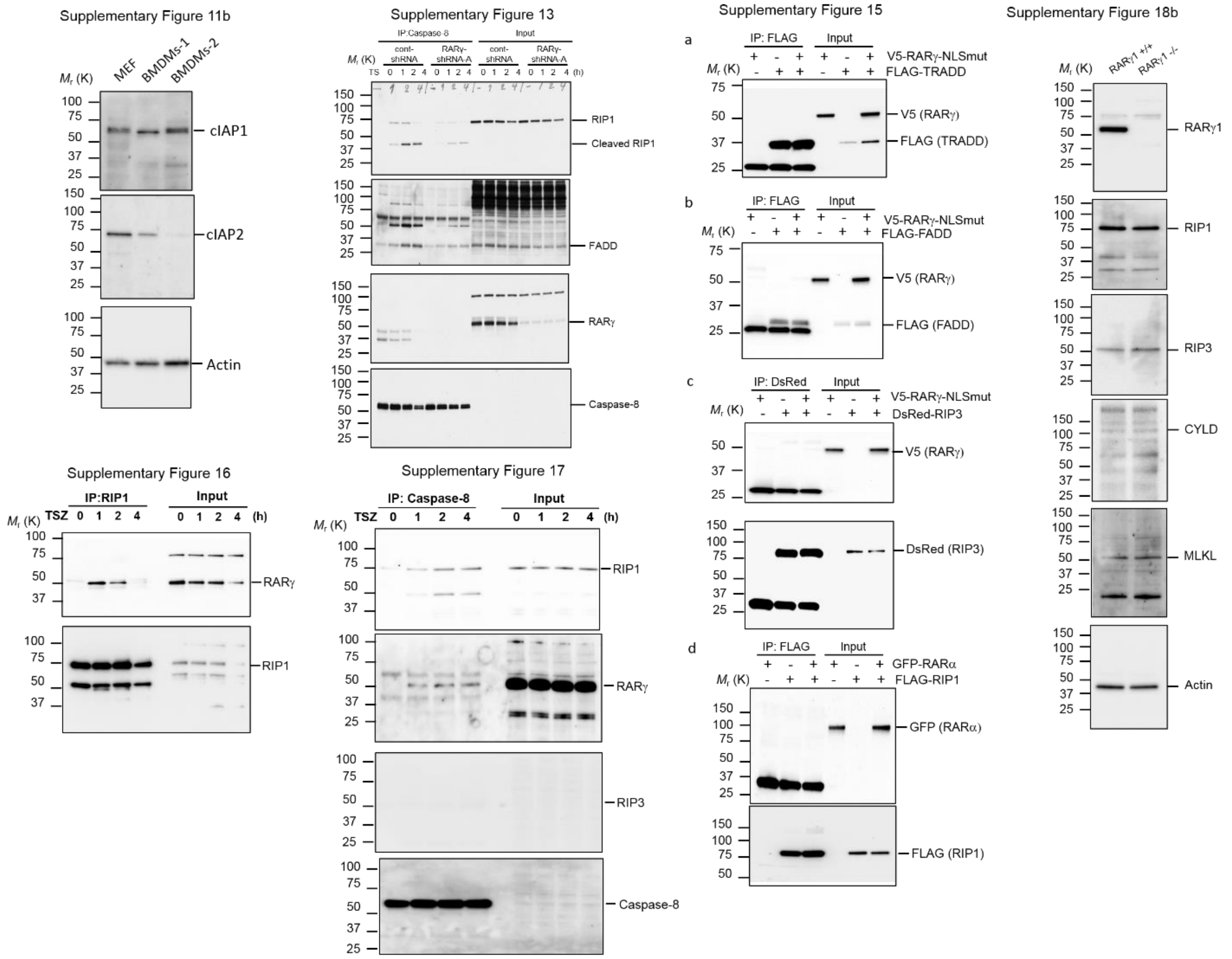

Supplementary Figure 29. Original immunoblotting of supplementary figures
